# Investigating a simplified method for noninvasive genetic sampling in East African mammals using silica dried scat swabs

**DOI:** 10.1002/ece3.6115

**Published:** 2020-03-06

**Authors:** Andrew J. Tighe, Sarah Overby, Kiera Thurman, Robert Gandola, Bernerd Fulanda, John Byrne, Jens Carlsson

**Affiliations:** ^1^ Area 52 Research Group School of Biology and Environmental Science/Earth Institute University College Dublin Dublin Ireland; ^2^ Fish Health Unit Marine Institute Oranmore Ireland; ^3^ Interdisciplinary Research Structure for Biotechnology and Biomedicine (ERI BIOTECMED) University of Valencia Valencia Spain; ^4^ Department of Biological Sciences Pwani University Kilifi Kenya

**Keywords:** DNA preservation, giraffe, impala, lion, oryx, scat

## Abstract

Swabbing scat has proved to be an effective noninvasive method to collect DNA from mammals in the field. Previously, this method has relied on preservative liquids or freezing to preserve the DNA collected on swabs. In this study, we determine the effectiveness of using silica to simply dry the swab in field as an alternative way to prevent DNA degredation. Four species were included in the study; reticulated giraffe, impala, fringe‐eared oryx, and lion. Swabs were taken at multiple time points for giraffe and impala scat samples, with the lion and oryx sampled opportunistically. Mitochondrial DNA was successfully amplified and sequenced from scat swabs from all species; however, effectiveness varied between species, with 81.8% amplification success rate from swabs taken from impala scat compared to 25% amplification success rate in giraffe. This variation in success rate was overcome by taking multiple swabs, thus increasing the probability of a successful amplification. The true merit of this method is in its simplicity and cheapness; no preservative liquids were required to be brought into the field, at no stage in the 2 weeks of field sampling were samples frozen, and no commercial kits were used for DNA extraction.

## Graphical abstract

This study has outlined how using silica to dry out scat swab samples in the field can be used as an alternative to using preservative liquids or freezing for preventing DNA degradation. The effectiveness of this technique was tested on giraffe, impala, oryx, and lion in the field in southeast Kenya.

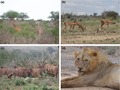

## 1 INTRODUCTION

Noninvasive DNA sampling provides a way to sample wild mammal populations without causing harm or stress to the target animal, while also being safer and logistically simpler for the researcher. In addition, it is often easier to obtain permits for noninvasive sampling than for studies that require physical interaction with the animal, in particular with threatened or endangered species. Sampling noninvasively implies a DNA collection method that does not require capturing or disturbing the target animal, with examples such as collecting hair (Rovang, Nielsen, & Stenhouse, 2015) to the more common approach of collecting and preserving scat (Hassanin, Ropiquet, Gourmand, Chardonnet, & Rigoulet, 2007; Masembe, Muwanika, Nyakaana, Arctander, & Siegismund, 2006; Silva et al., 2015; Tende, Hansson, Ottosson, & Bensch, 2014).

A number of studies have shown that swabbing scat in the field can be an effective method for collecting DNA samples for both carnivores (DeMatteo, Rinas, Argüelles, Holman, et al., 2014; DeMatteo, Rinas, Argüelles, Zurano, et al., 2014; Lampa, Gruber, Henle, & Hoehn, 2008; Miles, Holtz, Lounsberry, & Sacks, 2015; Ramón‐Laca, Soriano, Gleeson, & Godoy, 2015; Rutledge, Holloway, Patterson, & White, 2009) and herbivores (Ramón‐Laca et al., 2015; Renan et al., 2012; Tighe et al., 2018). However, most of these studies used either a preservative liquid (Ramón‐Laca et al., 2015; Renan et al., 2012; Rutledge et al., 2009) or froze the swabs within 24 hr (DeMatteo, Rinas, Argüelles, Holman, et al., 2014; DeMatteo, Rinas, Argüelles, Zurano, et al., 2014; Lampa et al., 2008; Renan et al., 2012). These preservative methods can be an issue when working in remote field sites, such as the site of this study. In such field sites, the facilities needed to freeze samples are often not available, and so researchers rely on preservative liquids such as ethanol or a lysis buffer. This in turn introduces another issue, which is if the samples have to be transported via aeroplane (which is often the case with samples from Africa), airport security may not permit liquids such as ethanol on the flight or may be suspicious of small vials of liquid. Some alternative methods have demonstrated how drying swab samples can be an effective means of preservation which circumvent the issues of freezing and liquids. Examples include Miles et al. (2015) in which mesocarnivore scat samples were collected in the field in paper bags and later swabbed with ethanol‐moistened cotton swabs, Quasim, MacDonald, and Sarre (2018) in which mammal predator scats were swabbed, air‐dried in the field, and then stored at room temperature, and a previous study by our laboratory group where African bush elephant (*Loxodonta africana*) scat was swabbed in the field and the swabs were preserved using silica (Tighe et al., 2018).

A range of factors can affect the success of amplifying DNA from mammalian scat, such as temperature (Hájková et al., 2006), moisture content and environmental humidity (Regnaut, Lucas, & Fumagalli, 2006; Vynne, Baker, Breuer, & Wasser, 2012), and diet (Maudet, Luikart, Dubray, Hardenberg, & Taberlet, 2004; Murphy, Waits, & Kendall, 2003). For example, herbivore scat can contain a higher amount of PCR inhibitors than carnivore scat due to the higher amount secondary metabolites from plant material (Espinosa, Bertin, Squeo, Cortés, & Gouin, 2015). This higher level of inhibitors may then affect which DNA extraction method works best for herbivore scat, with methods such as the cetyltrimethylammonium bromide (CTAB) protocol (Möller, Bahnweg, Sandermann, & Geiger, 1992) being more effective at separating DNA from cellular debris high in polysaccharides, as would be expected in the fecal matter of an herbivore (Espinosa et al., 2015). Espinosa et al. (2015) showed how the CTAB protocol can be more effective than a commercial QIAamp DNA Stool Mini Kit when extracting DNA from onager (*Equus hemionus*) scat samples.

Moisture has been shown to be a major factor in the degradation of DNA in scat, as it facilitates microbial and DNase activity (Regnaut et al., 2006; Vynne et al., 2012). By storing the scat swabs with silica, any moisture is removed, thus limiting biological activity which could degrade the target DNA. In this study, the method used for elephants by Tighe et al. (2018) of preserving scat swabs in the field using silica beads has been tested on scat samples from reticulated giraffe (*Giraffa reticulata*), impala (*Aepyceros melampus*), fringe‐eared oryx (*Oryx beisa callotis*), and lion (*Panthera leo*) to examine whether the method can be applied to a wider range of species. The giraffe and impala were the initial target animals with swabs taken from multiple time points after the initial defecation, and the lion and oryx were tested opportunistically following observed defecation. By using this method to dry the swabs, at no point in the field or during transport did the samples need to be frozen or placed in a preservative liquid. Additionally, due to the herbivorous diet of the target animals (with the exception of the lion) and to keep laboratory costs low, the CTAB protocol was used for DNA extraction.

## 2 MATERIALS AND METHODS

### 2.1 Sample collection

**Figure 1:**
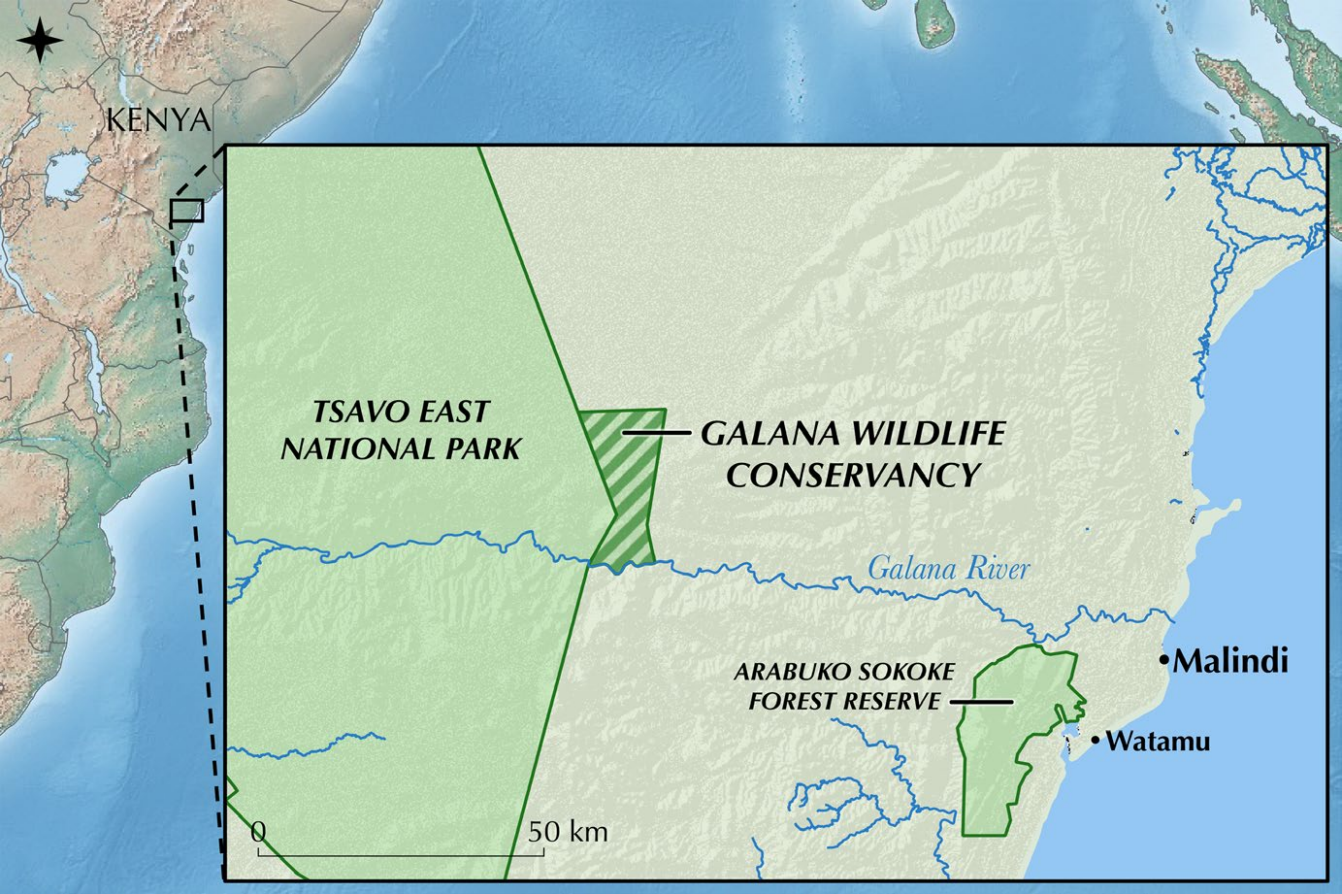
Map of study site. Map showing East Africa with inset of the study region in Kenya

Samples were collected from 18 May to 27 May 2017 in the Galana Wildlife Conservancy (GWC), which borders Tsavo East National Park in southeast Kenya (Figure 1). No fencing is present around the GWC allowing the free ranging of wildlife between the conservancy and Tsavo East National Park, with the main habitat present being semi‐arid scrub. Sighting surveys for the target animals were conducted in the morning (06:00–08:00 hr), and when an animal had been targeted, it was followed until defecation occurred. Once the animal had moved to a safe distance, sterile synthetic tipped swabs (FLOQSwabs, Copan) were dipped in phosphate‐buffered saline (PBS) solution (pH 7.4) and then rubbed on the outer surface of the scat sample until visibly covered in fecal material.

**Figure 2:**
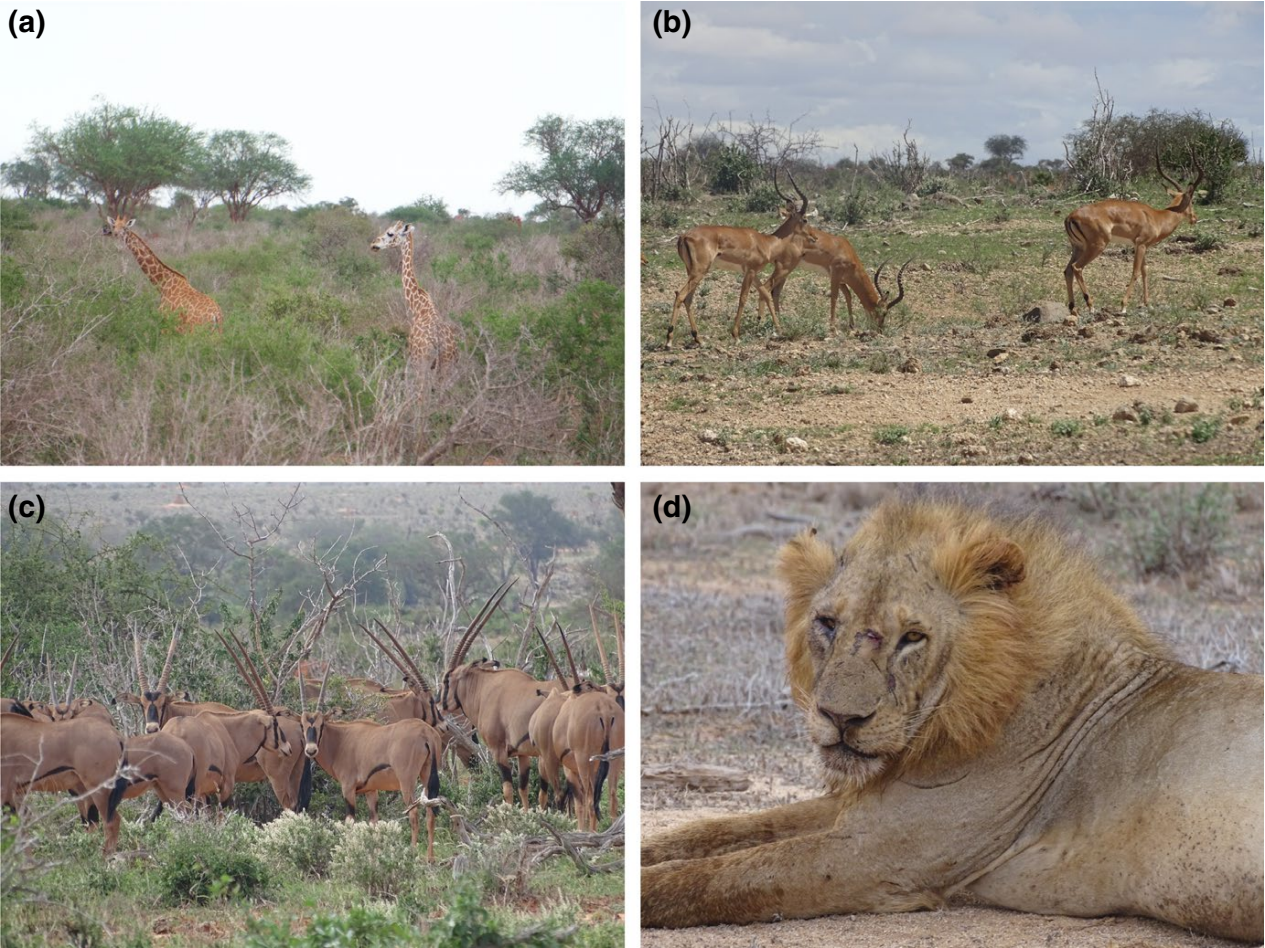
Species sampled. Pictures taken in the GWC of the target species involved in this study. (a) Reticulated giraffes. (b) Male impalas. (c) Fringe‐eared oryx herd. (d) Lion, showing the reduced mane common in Tsavo lions

Samples were taken from four species, reticulated giraffe, impala, lion, and fringe‐eared oryx (Figure 2 and Table 1). The giraffe species were identified based on coat patterns and median ossicone morphology, which were closer to what is typical in reticulated giraffes as opposed to Masai giraffes (*Giraffa tippelskirchi*). For the giraffe scat samples, two swabs were first taken when fresh and then swabbed again at multiple time points after 1, 2, 4, 8, 16, and 32 hr. For the impala samples, two swabs were taken when fresh and then at the same time points as the giraffe. For the lion sample, two swabs were taken when the sample was fresh, and the scat was then revisited after 64 hr and swabbed again. The oryx swabs were taken only from fresh samples, with no repeat sampling at different time points.

**Table 1:** Number of swabs taken for each target animal in this study

Species	Animal	Sex	Time						
			0−1 hr	2 hr	4 hr	8 hr	16 hr	32 hr	64 hr
*Giraffa reticulata*	A	♂	2^a^, 2^s^	1^a^, 1^s^	1^a^, 1^s^	1^a^, 1^s^	1^a^, 1^s^	1^a^, 1^s^	—
	B	**♀**	2^a^, 2^s^	1^a^, 1^s^	1^a^, 1^s^	1^a^, 1^s^	1^a^, 1^s^	—	—
*Aepyceros melampus*	A	**♀**	4^s^	2^s^	2^s^	1^s^	1^s^	1^s^	—
	B	**♀**	4^s^	2^s^	2^s^	2^s^	2^s^	2^s^	—
	C	**♀**	3^s^	1^s^	1^s^	1^s^	1^s^	1^s^	—
*Oryx beisa callotis*	A	—	1^s^	—	—	—	—	—	—
	B	—	1^s^	—	—	—	—	—	—
*Panthera leo*	A	**♂**	2^s^	—	—	—	—	—	1^s^

Abbreviations: a, air‐dried; s, silica dried.

After the scat swabs were taken, the tip of each swab was then cut off and placed into a 2‐ml O‐ring tube (Eppendorf) containing approximately six silica beads (2.5–6.0 mm) (Fisher Scientific). For the giraffe swab samples, half were air‐dried and placed in an O‐ring tube without silica beads as a comparison (Table 1). The O‐ring tubes were then further sealed with parafilm back at camp and were kept out of direct sunlight. At no point were the swab samples frozen while in the field or during transport, with average the average temperature in camp being ~30°C. Once back in the laboratory at University College Dublin, samples were kept at −20°C pending analysis.

### 2.2 DNA extraction

A modified version of the CTAB protocol was used for DNA extractions due to the likely presence of PCR inhibitors in the fecal samples (Ramón‐Laca et al., 2015). Each swab was placed in a lysis mix of CTAB and proteinase k (20 mg/ml) and left overnight at 56°C. This was followed by the addition of RNaseA and incubation of the mixture at 37°C for 30 min. DNA was extracted using chloroform–isoamyl alcohol and then precipitated using isopropanol and ethanol. A negative control consisting of purified water was included in each set of extractions. DNA concentration was quantified using a BioDrop μLITE spectrophotometer.

### 2.3 Mitochondrial amplification and sequence analysis

For each species, the mitochondrial DNA (mtDNA) region chosen for analysis was based on pre‐existing sequence on GenBank which would allow for adequate comparison to following sequencing. For giraffe samples, a 583‐bp segment of the cytochrome b (*Cytb*) gene was amplified using primers F (5′‐TGA AAA ACC ATC GTT GTC GT‐3′) and R (5′‐GTG GAA GGC GAA GAA TCG‐3′) (Bock et al., 2014). A 20 µl PCR master mix was prepared in a UV‐sterilized hood and consisted of 2 µl 10X Buffer (Kapa Biosystems), 0.4 μl dNTP (Invitrogen), 0.08 Taq polymerase (Kapa Biosystems), 0.8 μl of each primer (10 μM) (Integrated DNA Technologies), 14.42 μl water, and 1.5 μl DNA (undiluted). PCR conditions were as follows: initiation at 95°C for 5 min, followed by 35 cycles of 95°C for 30 s, 50°C for 30 s, and 72°C for 1 min. The final elongation step was 72°C for 5 min, after which samples were held at 4°C.

For impala samples, a 555‐bp segment of the NADH dehydrogenase subunit 4 (*NAD4*) gene was amplified with the primers F (5′‐CCT ACC CCT GTT AGT CGC AC‐3′) and R (5′‐TAT AGT CCG GCT GTG GAT GC‐3′). A 15 μl PCR master mix was prepared in a UV‐sterilized hood and consisted of 1.5 μl 10X Buffer, 1.5 μl dNTP, 0.15 Taq polymerase, 0.3 μl of each primer (10 μM), 9.75 μl water, and 1.5 μl DNA (undiluted). PCR conditions were as follows: initiation at 95°C for 5 min, followed by 35 cycles of 95°C for 30 s, 50°C for 30 s and 72°C for 1 min. The final elongation step was 72°C for 5 min, after which samples were held at 4°C.

For oryx samples, a 1049‐bp segment of *Cytb* was amplified using primers CBU162 (5′‐CAG GMC TAT TCC TRG CHA TAC A‐3′) and LTHR (5′‐CCC TTY TCT GGT TTA CAA GAC C‐3′) (Hassanin, Ropiquet, Couloux, & Cruaud, 2009). A 30 μl PCR master mix was prepared in a UV‐sterilized hood and consisted of 3 μl 10X Buffer, 3 μl dNTP, 0.15 Taq polymerase, 1.5 μl of each primer (10 μM), 2 μl BSA (10 mg/ml) (Thermo Fisher Scientific), 16.85 μl water, and 2 μl DNA (undiluted). PCR conditions were as follows: initiation at 94°C for 3 min, followed by 35 cycles of 94°C for 1 min, 50°C for 1 min, and 72°C for 1 min. The final elongation step was 72°C for 7 min, after which samples were held at 4°C.

For lion samples, a 488‐bp segment of *Cytb* was amplified using primers 1F (5′‐CGT TGT ACT TCA ACT ATA AGA ACT T‐3′) and 1R (5′‐ATG GGA TTG CTG ATA GGA GAT TAG‐3′) (Bertola et al., 2011). A 20 μl PCR master mix was prepared in a UV‐sterilized hood and consisted of 2 μl 10X Buffer, 0.4 μl dNTP, 0.08 Taq polymerase, 0.8 μl of each primer (10 μM), 1.5 μl BSA (10 mg/ml), 13.42 μl water, and 1 μl DNA (undiluted). PCR conditions were as follows: initiation at 94°C for 4 min, followed by 60 cycles of 93°C for 20 s, 55°C for 30 s, and 72°C for 30 s. The final elongation step was 72°C for 10 min, after which samples were held at 4°C. All PCR amplifications were undertaken in a separate room from where DNA was extracted. For all amplifications, a negative control was included which consisted of purified water.

Post‐PCR, samples were subject to electrophoresis on a 1% agarose gel, in a separate room again from where amplification took place, and a successful amplification was based on the presence of a band of the correct molecular weight in the gel. Positive samples were then sequenced commercially via Sanger sequencing (Macrogen). Forward and reverse strands were aligned using Geneious version 10.2.3 (Kearse et al., 2012). The resulting consensus sequences were then analyzed using BLAST (https://blast.ncbi.nlm.nih.gov/Blast.cgi). The sequences from this study were then aligned with closely related sequences from Genbank using ClustalX2 (Larkin et al., 2007). The aligned sequences were then subject to a model test using MEGA7 (Kumar, Stecher, & Tamura, 2016), and following this, MEGA7 was used to generate neighbor‐joining trees for each animal, with 1,000 bootstrap replicates performed in order to test the robustness of the phylogeny generated.

## 3 RESULTS

### 3.1 Amplification success

**Figure 3:**
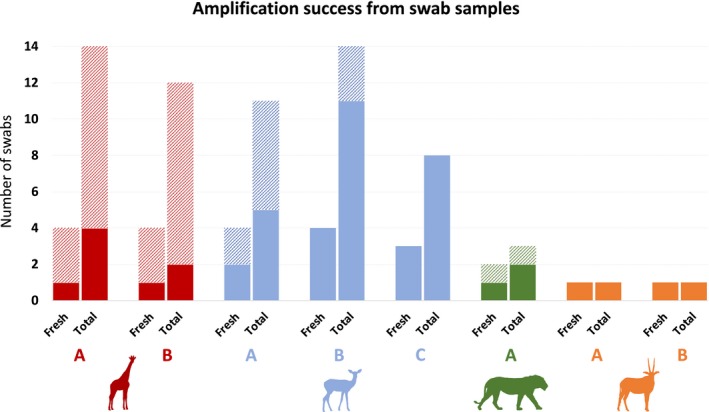
Amplification success rate from swabs from mammal species in this study. For each animal, success rate from both the fresh (taken within 2 hr) and total swabs is shown. Color indicates species; red = giraffe, blue = impala, green = lion, and orange = oryx. Full color indicates a successful amplification, and lighter color indicates an unsuccessful amplification

Target DNA was successfully amplified for all animals at least once from a scat swab (Figure 3). The giraffe samples showed the lowest amplification success rate, namely 25% from samples taken within the first hour and 23% overall. The lion samples amplified from 50% of the samples from the first hour and 67% overall. For the impala samples, successful amplification was seen in 82% of samples from the first hour, dropping to 73% overall. For the oryx samples, 100% amplification was observed, but this was from a single scat sample for each animal.

One amplified sample from each animal was sent for commercial Sanger sequencing. Half of the giraffe swab samples were air‐dried and stored without silica for comparison and of the giraffe scat samples sent for sequencing, both were from fresh scat with one having been preserved in silica beads (giraffe A) and the other from a sample stored without silica (giraffe B). For impala and oryx, all samples sent for sequencing were taken from fresh samples. For lion, the sample sent for sequencing was from the swab taken when the scat was 64 hr old.

### 3.2 Sequence analysis

A 566‐bp sequence was generated for giraffe A, which matched 99.11% (556/561bp) to a reticulated giraffe sequence collected from Sigean African Reserve, France (Hassanin et al., 2007). The chromatogram for this sequence revealed contamination of the sequence data for this sample near both primer ends, which BLAST revealed to be sequence from a species of *Masillia* bacteria, which is found in soil (Lou, Gu, Wang, An, & Xu, 2016). The overlapping sequence from the opposite strands however allowed for a consensus sequence to be generated. All other sequences generated were free of contamination.

For giraffe B, a 500‐bp sequence was generated, which matched 100% to 11 sequences on Genbank from reticulated giraffe and one from Masai giraffe*,* with samples originating in Kenya, Tanzania, and two zoos in Europe (Brown et al., 2007; Fennessy et al., 2016; Hassanin et al., 2007; Winter, Fennessy, Fennessy, & Janke, 2018). In the case of the match to Masai giraffe, this was to a sequence (EU088334) which came from a study by Brown et al. (2007) that found a paraphyletic haplotype for Masai giraffe that was clustered within reticulated giraffe mtDNA haplotypes, which may explain the single match.

For the impala samples, a 448‐bp sequence was generated for impala A, a 426‐bp sequence for impala B, and a 451‐bp sequence for impala C. All three matched (A: 99.11%, B: 99.3%, C: 99.11%) to a single impala sequence, which was collected in Tanzania (Hassanin et al., 2012). Over the 424bp where all three sequences overlap, there were three variable sites, with A and C being identical.

A 475‐bp sequence was generated for the lion sample, which matched 99.79% to three lion sequences on Genbank, which had been collected in Zambia, Somalia, and Kenya (Bertola et al., 2016).

For the oryx A, a 976‐bp sequence was generated that matched 99.79% to a fringe‐eared oryx sample which had been collected in Burko forest reserve, Tanzania (Masembe et al., 2006). Additionally, when compared to a sequence collected by Masembe et al. (2006) from Tsavo East, Kenya, the sequence matched 99.47%. A 974‐bp sequence was generated for oryx B which matched 99.9% to a fringe‐eared oryx sample from Samburu National Reserve, Kenya (Masembe et al., 2006). In addition, when compared to the sequence from Tsavo East, oryx B matches 99.27%. The two oryx samples share 99.38% similarity over the 969bp they overlap (963/969bp).

All sequences were submitted to GenBank (accession numbers: MN999547–MN999553), with exception of giraffe A due to the observed contamination.

## 4 DISCUSSION

As population and conservation studies become increasingly dependent on collecting genetic data from target species, there is a coinciding need for the development of simple methods for DNA collection. In this study, we have outlined a novel method which is both simple and cheap using only silica as a preservative, and we have shown its applicability to both herbivores and carnivores. DNA samples were obtained for all target animals; however, the efficiency of the method varies between species and seems to be of limited effectiveness for scat samples from giraffes. A similar study by Renan et al. (2012) in which various methods of preserving scat DNA were tested found a 50% amplification success for mitochondrial DNA from swabs taken from fresh onager (*Equus hemionus*) scat which were stored on ice in the field and frozen at −20°C within a few hours and extracted using the CTAB method. In our study, we observed 25% amplification success from fresh giraffe scat and 81.8% success from fresh impala scat, suggesting our method to be more effective when using impala scat, while less effective when using giraffe scat. Due to the very small sample size for the lion and oryx samples, we refrain from comparison. It should be noted also that Renan et al. (2012) took samples from a larger number of individual animals (*n* = 24) and thus may account for more variation between individuals than in our study. Additionally, Renan et al. (2012) observed 100% amplification success when using QIAamp DNA Stool Mini Kit (QIAGEN) instead of the CTAB method. For our method, however at no stage in the field were samples cooled or frozen and were stored at ~30°C for the duration of fieldwork (2 weeks for samples taken at the beginning of the fieldwork) and likewise at no point during transportation back to Ireland were samples frozen. Only when back in the laboratory in Ireland were samples placed in the freezer.

Clean unambiguous sequence chromatograms were obtained for all four species, with the exception being the sequence data for one of the giraffe chromatograms which had been contaminated with bacterial DNA. The consensus sequence obtained from both giraffes supports the initial species assignment as reticulated giraffes; however, as only one mtDNA locus was examined it cannot be ruled out that these animals are hybrids between reticulated and Masai giraffes, as suggested by their intermediate morphology. In addition, the area between the Galana River and the Tana River has been described as an intergrade between the two species (East, 1999) adding to the possibility of hybridization.

**Figure 4:**
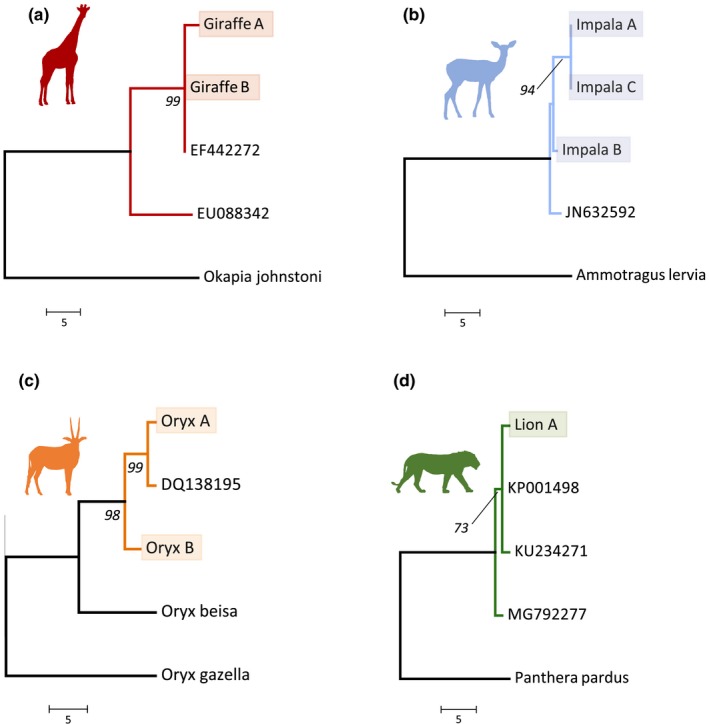
Neighbor‐joining phylogenetic trees showing the relationship of the sequences generated in this study to closely related sequences from Genbank. The sequences generated in this study are highlighted. (a) Tree showing the giraffe sequences using Okapi (*Okapia johnstoni*) as an outgroup. (b) Tree showing the impala sequences using Barbary sheep (*Ammotragus lervia*) as an outgroup. (c) Tree showing oryx sequences using both common beisa oryx (*Oryx beisa*) and gemsbok (*Oryx gazella*) as outgroups. (d) Tree showing lion sequence using leopard (*Panthera pardus*) as an outgroup

For the other species in the study, the sequence data revealed a previously unrecorded haplotype for lion, two unrecorded haplotypes for impala, and two unrecorded haplotypes for fringe‐eared oryx (Figure 4). The two fringe‐eared oryx sequences were more closely related to sequences from Samburu National Reserve, Kenya, and Burko forest reserve, Tanzania, than they were to sequences collected from the neighboring Tsavo East National Park. This may suggest that the oryx populations in all three locations are part of one large population which may have implications for conservation efforts. In addition, these two new oryx sequences add to the growing molecular evidence, in addition to morphological evidence, that the fringe‐eared oryx subspecies is in fact a separate species (*Oryx callotis*) from the common beisa oryx (*Oryx beisa beisa*) as originally described by O. Thomas (1892) (Groves & Grubb, 2011; Lee, Dolman, & Leslie, 2013; Mahato & Raziuddin, 2012).

In conclusion, this study has outlined a novel noninvasive method for collecting mtDNA samples from scat from various East African mammals, both herbivores and a carnivore species. Ideally, we would have included samples from more individuals from each species to lessen the probability of sampling a low‐quality scat, but performing fieldwork on a limited timescale in a challenging environment places restrictions on what can be achieved in projects such as this. While the effectiveness of the method can vary between species as seen by the amplification success, we argue that the true merit of this method is in its simplicity; no preservation liquids were used at any stage, the samples did not need to be frozen at any point in the field, and the extraction method of using CTAB is cheaper than using a commercially available kit. In order to overcome variability in amplification success, we recommend future studies that want to try this method take multiple swab samples from the target animal's scat, and from as many individuals as possible, increasing the probability of amplification success.

## CONFLICT OF INTEREST

The authors declare no competing interests.

## AUTHOR CONTRIBUTIONS

A.J.T., S.O., K.T., J.B., and J.C. devised the study. A.J.T., S.O., K.T., and J.B. performed the fieldwork. B.F. facilitated the fieldwork and sampling. A.J.T., S.O., K.T., and R.G. performed the laboratory work and analysis, with additional support from B.F. and J.C. A.J.T. drafted the manuscript, figures, and tables. All authors contributed to writing and improving each manuscript draft, and all approved the final submitted draft.

## Data Availability

The DNA sequences generated in this study are available on NCBI GenBank under the accession numbers MN999547–MN999553.
